# “I feel I have no voice”: hospital doctors' workplace silence in Ireland

**DOI:** 10.1108/JHOM-08-2020-0353

**Published:** 2021-05-07

**Authors:** Jennifer Creese, John-Paul Byrne, Anne Matthews, Aoife M. McDermott, Edel Conway, Niamh Humphries

**Affiliations:** Royal College of Physicians of Ireland , Dublin, Ireland; School of Nursing, Psychotherapy and Community Health, DCU , Dublin, Ireland; Cardiff Business School, Cardiff University , Cardiff, UK; DCU Business School, Dublin City University , Dublin, Ireland

**Keywords:** Silence, Working conditions, Health workforce, Doctors, Ireland

## Abstract

**Purpose:**

Workplace silence impedes productivity, job satisfaction and retention, key issues for the hospital workforce worldwide. It can have a negative effect on patient outcomes and safety and human resources in healthcare organisations. This study aims to examine factors that influence workplace silence among hospital doctors in Ireland.

**Design/methodology/approach:**

A national, cross-sectional, online survey of hospital doctors in Ireland was conducted in October–November 2019; 1,070 hospital doctors responded. This paper focuses on responses to the question “If you had concerns about your working conditions, would you raise them?”. In total, 227 hospital doctor respondents (25%) stated that they would not raise concerns about their working conditions. Qualitative thematic analysis was carried out on free-text responses to explore why these doctors choose to opt for silence regarding their working conditions.

**Findings:**

Reputational risk, lack of energy and time, a perceived inability to effect change and cultural norms all discourage doctors from raising concerns about working conditions. Apathy arose as change to working conditions was perceived as highly unlikely. In turn, this had scope to lead to neglect and exit. Voice was seen as risky for some respondents, who feared that complaining could damage their career prospects and workplace relationships.

**Originality/value:**

This study highlights the systemic, cultural and practical issues that pressure hospital doctors in Ireland to opt for silence around working conditions. It adds to the literature on workplace silence and voice within the medical profession and provides a framework for comparative analysis of doctors' silence and voice in other settings.

## Introduction

Organisational voice and silence are of vital importance in healthcare settings, impacting staff experiences, organisational capabilities and patient safety ( Dixon-Woods *et al.* , 2019 ; Martin *et al.* , 2018 ; Wilkinson *et al.* , 2015 ). The right for health workers to express concerns about working conditions is enshrined in the Conventions of the International Labour Organisation ( Wiskow, 2017 ). Learning from the voices of frontline staff is key for responsive health services and competitive healthcare organisations and is particularly important in the context of the COVID-19 pandemic where healthcare systems and organisations need to act and learn as fast as possible ( Bohmer *et al.* , 2020 ; Daphna-Tekoah *et al.* , 2020 ). However, studies of health workers across the world show that their concerns can often go unheard or unexpressed ( Hughes, 2019 ; Landgren *et al.* , 2016 ; Lock *et al.* , 2017 ). This research builds on existing work on organisational voice in a healthcare context, examining the reasons why hospital doctors in Ireland are reluctant to voice their concerns about their working conditions. Importantly, voice and silence are distinct behaviours, such that enablers of voice may not be sufficient to overcome silence ( Knoll and Redman, 2016 ). This has resulted in calls for research to focus on why employees withhold their voice and engage in silence.

This study sheds light on the organisational, professional and cultural factors contributing to that silence in Irish hospital medicine and provides insights that should be relevant to hospital medicine in other country contexts. Previous studies of doctors in Ireland and Irish emigrant doctors overseas have touched on issues of voice in relation to unfair treatment ( Crowe *et al.* , 2017 ) or in the use of exit over voice ( Humphries *et al.* , 2019 ). This paper builds on the issues these studies introduce, offering a more detailed analysis of doctors' silence in the Irish setting, with wider relevance to studies of silence on working conditions in health outside Ireland. Reducing silence and enhancing voice mechanisms may help the Irish health system improve learning and support its workforce and reduce the likelihood of emigration and burnout.

The Republic of Ireland operates a two-tiered public–private health system, with the delivery of public health services under the remit of the Health Service Executive (HSE). Ireland's public health system has been severely strained and underfunded for decades, particularly since the global financial crisis of 2008 and subsequent austerity measures, which have cut public service funding ( Burke *et al.* , 2014 ; McGowan *et al.* , 2013 ; Turner, 2018 ). This has resulted in deteriorating job quality for hospital doctors: reduced resourcing, heavier workloads and longer working hours. In turn, these issues have driven high rates of doctor emigration ( Brugha *et al.* , 2016 ; Humphries *et al.* , 2019a , b ). Amongst those doctors who remain in Ireland, burnout has been found to be high ( Hayes *et al.* , 2019 ; McNicholas *et al.* , 2020 ; O'Dea *et al.* , 2017 ). At the time of writing, the Irish health system also faces significant changes and challenges due to the introduction of the Sláintecare healthcare system reforms scheduled for the 2018–2028 period ( Burke *et al.* , 2018 ) and, though after the data collection period of this research, the COVID-19 pandemic ( Kennelly *et al.* , 2020 ).

## Workplace silence and voice

In organisational literature, silence has a variety of different definitions and interpretations. Silence can be understood at an organisational or group level, stemming from a systemic lack of formal voice mechanisms within an organisation ( Morrison and Milliken, 2000 ), a workplace culture of contempt for voice ( Morrison and Milliken, 2000 ; Wilkinson *et al.* , 2015 ) or poor “psychological safety”, the belief that the work environment is unsafe for personal risk-taking ( Edmondson, 2019 ). Ineffective or lack of organisational change can cause a “deaf effect” ( Jones and Kelly, 2014 ; Martin *et al.* , 2018 ) where voices calling for change are neither heard nor actioned and the status quo remains.

Silence can also be understood on a personal, individual level, as a conscious withholding of voice from the organisation by an individual employee ( Pinder and Harlos, 2001 ; Van Dyne *et al.* , 2003 ). Silence may be driven by apathy, labelled “acquiescent silence” ( Pinder and Harlos, 2001 ; Van Dyne *et al.* , 2003 ), a passive form of silence where voice is desired by workers but not expected to bring about change, leading to disengagement. Silence may also stem from fear, labelled “quiescent” ( Pinder and Harlos, 2001 ) or “defensive” ( Van Dyne *et al.* , 2003 ) silence, where the individual employee perceives negative consequences for themselves if they speak out. This is an active form of silence, where risks of using voice are consciously weighed and silence chosen as the best personal strategy.

Silence is shaped by a range of contextual and individual factors that inform how workers perceive the efficacy and safety of speaking out or remaining silent ( Morrison, 2011 ; Withey and Cooper, 1989 ). Working lives are mediated by a range of “conversion factors” ( Hobson, 2013 ) that enable or constrain workers' agency: societal and regulatory factors like laws or socio-cultural norms (macro), organisational and departmental factors like policies and institutional norms (meso) and individual dispositions, beliefs and domestic factors like family or financial needs (micro) ( Wilkinson *et al.* , 2018a , b ). These “conversion factors” and forms of “capital” come together in different ways for different workers; some are silent and have limited opportunities to use their voices to achieve the change they desire, while others are enabled and empowered to use their voices for change. Factors causing silence and enabling voice may also overlap for some individuals ( Knoll and Redman, 2016 ).

The concept of voice has been identified and characterised in a variety of ways across organisational literature. Voice can be understood as discretionary, informal communication upwards within the organisation, aiming to bring about change when employees are dissatisfied ( Hirschman, 1970 ; Morrison, 2011 ). Hirschman positions voice alongside exit and loyalty as options for dissatisfied employees; Rusbult *et al.* (1982) add neglect to this list of options. Voice is also understood as a system-level phenomenon, rather than an individual one; the process through which employees make actions and appeals for change, and contribute to organisational decision-making, through the employer or a union ( Pyman *et al.* , 2006 ; Wilkinson *et al.* , 2004 ; Wilkinson *et al.* , 2018a , b ). In justice terms, voice is understood as the provision of testimony or whistleblowing to uncover serious organisational problems ( Kenny, 2019 ; Pinder and Harlos, 2001 ).

Good voice experiences – being able to raise concerns at work and have them actioned with no negative repercussions – are linked positively to employees' commitment, engagement, sense of belonging, self-esteem and job satisfaction ( Edmonson, 2009 ; Morrison and Milliken, 2000 ; Ryan and Oestreich, 1991 ). Working without effective mechanisms to voice concerns can negatively influence psychological and physiological well-being ( Morrison and Milliken, 2000 ). Conversely, silence, stemming from an inability to safely raise concerns at work and have them effectively actioned, has been shown to render employees less motivated, satisfied and engaged at work, less adaptable to organisational change and less committed to remain part of the organisation ( Morrison and Milliken, 2000 ; Ryan and Oestreich, 1991 ). Ultimately, silence can generate apathy, making employees more likely to exit their organisation or system, or to be neglectful of themselves or their work, calling in sick or late, losing interest, making errors and processing problems poorly ( Ryan and Oestreich, 1991 ; Withey and Cooper, 1989 ).

## Silence and voice in the healthcare context

Voice has a unique dimension in the healthcare context: as Wilkinson *et al.* (2020 , p. 562) state, “any given concern or issue a healthcare worker has may relate specifically to patient care or their own well-being and working conditions”. Healthcare workers, including doctors, have been actively trained and encouraged to voice concerns directly related to patient care (see, e.g. Ward *et al.* , 2019 ), and most reportedly will do so ( Landgren *et al.* , 2016 ; Schwappach and Richard, 2018 ). Much of the literature on voice in the healthcare context focuses on voice in patient welfare ( Wilkinson *et al.* , 2020 ). However, doctors are often silent when it comes to issues relating to their own professional conditions ( Martinez *et al.* , 2017 ; Wilkinson *et al.* , 2020 ). The health sector has begun to take the turn towards “fostering climates and cultures of respect—in part encouraged by the realization that high-reliability organizations in other sectors do likewise” ( Dixon-Woods *et al.* , 2019 , p. 579), but progress has been slow and difficult.

While, on paper, many hospitals have adopted staff voice mechanisms ( Wilkinson *et al.* , 2018a , b ), in practice, poor implementation of staff suggestions or “organisational deafness” has been identified as rife in healthcare settings ( Attree, 2007 ; Pope and Burnes, 2013 ; Wilkinson *et al.* , 2018 ), leading to apathy amongst doctors. Avgar *et al.* (2016) suggest that hospitals where doctors and administrators work collaboratively are more likely to have less silence. Yet, the cultural divides between doctors and administrators are widespread in contemporary hospitals, which create a barrier to engagement and collaboration ( Keller *et al.* , 2019 ). Voice and union effectiveness in hospitals are also closely linked ( Avgar *et al.* , 2016 ), yet Lipset *et al.* (2004) , studying professional union participation in both Canada and the USA, suggest that physicians are largely reluctant to join unions or use them for industrial action as they consider them “unprofessional”.

Professional culture in medicine is a key factor influencing silence around working conditions. The medical profession sets norms that govern professional identity, conduct and belonging, which are communicated to new entrants to the profession via the formal and “hidden curriculum” ( Mahood, 2011 ; Monrouxe, 2010 ). Several international studies of medical culture argue that the expectation of silence about negative work experiences is central to the “hidden curriculum” within medicine ( Helmreich and Merritt, 1998 ; Wear and Zarconi, 2010 ). Professional medical culture may also be seen to constitute a “field” in the sense of Bourdieu (1982) , where those at the core with high cultural and social capital set and emphasise norms, while those on the periphery with less cultural and social capital have less legitimacy in the field ( Anheier *et al.* , 1995 ). In medicine, the stratified power structures and power bases within the field dictate who does not have the “capital” to be able to use voice safely – those lower in the professional hierarchy, women and those from overseas ( Luke, 2003 ).

The health sector, then, has several factors that foster different reasons and outcomes for silence than other sectors. It is governed by powerful cultural forces and values, often dissociated from the administrative structures that manage doctors as workers. Unlike in other sectors, the nature of healthcare work is also not focused on productivity or economic output, but on treating all patients to the extent of their abilities and treatments. The outcomes of silence – exit, neglect, workplace error, reduced engagement and adaptability, and poor well-being – are of particular significance in healthcare. However, while these outcomes have been well documented and publicised, comparatively little attention has been paid to the *reasons* why doctors remain silent. This prompted the focus in this paper on those respondents who would not voice concerns about working conditions and exploration of the factors behind this decision.

## Method

This paper analyses voice-related closed and open-ended questions from a national cross-sectional survey of hospital doctors in Ireland. Using the Qualtrics online survey tool, the survey was distributed to a grade-stratified sample of 5,356 hospital doctors via the national Medical Register, with the assistance of the Medical Council of Ireland (for more information on methods, see Humphries *et al.* , 2020 ). An overall response rate of 1,070 responses (20%) was achieved – in line with similar surveys of hospital doctors ( O'Brien *et al.* , 2019 ; Shanafelt *et al.* , 2019 ) – which was reduced to 990 after filtering out incomplete responses. The survey included a mix of quantitative and open-response qualitative questions. In total, 895 respondents answered the question “If you had concerns about your working conditions, would you raise them?”; 450 stated they would, 227 stated they would not and 208 were unsure.

**Figure 1:**
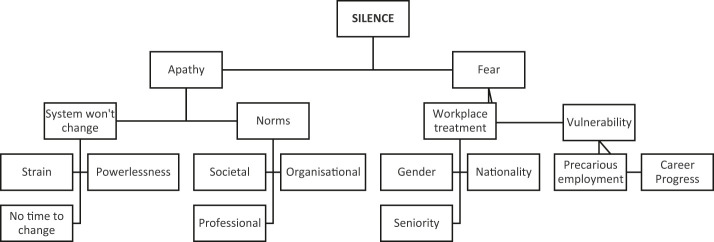
Themes and sub-themes from open-ended responses to question “Why would not you raise those concerns?”

Survey respondents who answered that they would not raise concerns about their working conditions were given a follow-up open-text question asking them to elaborate why not. Respondents who answered “yes” or “unsure” to raising working conditions were not prompted to elaborate. In total, 200 “no” respondents elaborated on their responses, and these were analysed utilising a thematic textual analysis approach ( Braun and Clarke, 2006 ). This is a method used effectively in other mixed methods studies in health services research ( Humphries *et al.* , 2015 , 2020 ; Popping, 2015 ). A combination of inductive and deductive coding was employed ( Fereday and Muir-Cochrane, 2006 ). Deductive themes of apathy and fear (i.e. acquiescent and quiescent silence) were generated, in accordance with Pinder and Harlos (2001) . Broader inductive themes and subthemes were also identified in the responses and coded ( Figure 1 ). Coding was completed by the first author and revised through iterations. Verbatim quotes from respondents are used throughout the paper, and respondents are referred to as *R* (Respondent), XXX (survey number), followed by their grade (i.e. Intern, Senior House Officer (SHO), Registrar, Specialist Registrar (SpR), Consultant).

The 227 respondent doctors who would not voice concerns about their working conditions (i.e. silent doctors) came from all levels of seniority and from all backgrounds. Table 1 shows a demographic profile of these by gender, country of citizenship, country of qualification, grade and locum status.

“Working conditions” were not explicitly defined in the survey and were open to respondents' own interpretations. The majority of respondent doctors did not specify any particular “working conditions” they had concerns about in their free-text responses; specific issues and conditions raised by some respondent doctors included staffing and health service funding levels, working hours, conflict between healthcare workers and conflict with hospital administration and the HSE systems. These align with working conditions that hospital doctors in Ireland, and expatriate Irish doctors in other countries, have identified as problematic in previous studies ( Humphries *et al.* , 2015 ; Humphries *et al.* , 2019a , b ; McGowan *et al.* , 2013 ).

## Findings

### Apathy: the improbability of change

Among silent doctors, silence driven by apathy was more common than fearful silence. In the 200 text responses analysed, there were 140 apathetic responses and 78 fearful responses; 18 respondents gave multiple reasons both apathetic and fearful for their silence. Apathetic respondents saw no personal risk inherent in speaking out, but no potential for change. They had little hope that the hospital or health system, already under strain, was likely to change:

Management are well aware of these concerns, and yet have no willingness to do anything about them. Raising them again would not be of benefit (R334, Consultant).

Raising concerns about lack of resources in the HSE would be like raising my concern about global warming (R165, Intern).

Some felt powerless in effecting change:

It's easier to get on with it than put time and effort into changing things that are not in my power to change (R391, SpR).

[I'm] not sure that it would help or that anybody would listen to me (R357, Intern).

Respondents also indicated that from a practical point of view, their heavy workloads left them with a limited amount of time to push for improved working conditions:

I do not have the time or energy to try and promote that change on top of my full-time job (R39, Intern).

There's no time in the working week to fight for change (R153, SHO).

### Normalising silence

Culture heavily influenced the silence of many respondent doctors. This was reported as a mix of an Irish cultural attitude towards complaint, a feature of the organisational culture of the health system, and a professional culture within medicine, all of which encouraged a silent acceptance of working conditions:

[There's] an Irish attitude of “get on with it” (R969, SpR).

Lots of talking but no action, no political will. It's the Irish way unfortunately (R987, Intern).

Raising these concerns achieves no positive outcome. It is considered the “norm” in most…hospitals in Ireland (R386, SHO).

For new entrants, messaging about voice norms came from their peers and superiors:

I've had multiple doctors, of various levels, express that “this is what working as a doctor is” (R57, Intern).

[There's an] atmosphere of we went through this so you should too. Feels like a “rite of passage” (R365, Intern).

### Silence is safer for the vulnerable

For many hospital doctors, career stage and grade were important factors in dictating whether they considered it appropriate to raise concerns about their working conditions. Many non-consultant hospital doctor respondents were reluctant to speak up about working conditions out of concern for the risk to their career. These doctors are employed on temporary six-month contracts until they progress to consultant grade, which takes a minimum of nine years. Many felt that they lacked the security and seniority to voice concerns and make change happen:

NCHDs are essentially told to put up with the way things are (R207, SpR).

Junior docs are powerless…nobody wants to hear it, consultants do not support [us] (R188, SHO).

Interns felt particularly reluctant to voice concerns. They remained silent because they were new to the hospital and to the realities of life as a hospital doctor, but also because of their lack of seniority. Some established these voice norms themselves:

As an intern I am new and feel I do not deserve to [speak up] yet because I have not worked as long as others (R58, Intern).

I do not feel comfortable as an intern asking a locum consultant [or] complaining to [a] registrar (R142, Intern).

Non-consultant hospital doctors were highly conscious of the reputational risks of voice, and often saw speaking up as a career risk outweighing any benefit they might gain.

It would risk earning me a reputation as being difficult to work with (R183, Reg).

I would be known as someone who caused a problem (R1014, Intern).

Non-consultant hospital doctors felt like they needed good references from senior hospital doctors to progress their careers and faced a risk of stalled or blocked career progression if they voiced concerns:

The consultants would not be happy [with me] and I need their reference (R785, SHO).

Respondents pointed out that as a small country, Ireland has a relatively small medical profession who are highly interconnected. This can be beneficial for training and collegiality but heightens reputational concerns and may discourage voice:

In a system as small as Ireland, the consultants you complain to will be the same ones writing your references [or] sitting on the [training] interview panels, [it's] better and wiser to just put up with it (R354, SHO).

Some female doctors argued that speaking out would change nothing and would attract the sort of negative gendered stereotyping that might damage their professional reputation:

I would worry about being seen as a nag or troublemaker (R779, Female SpR).

[I] could be labelled as difficult (R85, Female SpR).

Some non-European doctors felt that not being Irish citizens made them outsiders who would be less likely to be listened to:

I've been told Irish people protect each other and [if I report problems] in the end I'll be the laughing-stock (R462, SHO).

As a non-EU citizen… I feel I have no voice and my work and abilities [are] not recognised (R879, Reg).

In addition, some non-European doctors felt that speaking out might damage their reputations, career progression and job security:

As I'm a non-Irish doctor the administration does not care about my concerns, they feel like I'm easy to replace (R410, Reg).

I need to have my contract and work permit renewed every 6 months [so] I do not want to be seen as being difficult or not hardworking (R553, SHO).

### Implications of perceived need for silence

When lacking the desire or ability to voice, respondents were left to consider their options. For many, there was little choice but to continue working on in silence, blocking out any concerns about their working conditions:

Everyone has the same concerns, it's just [become] white noise (R158, Intern).

This is tolerable when you know you are only going to be in a centre for a year max… disincentive to change (R171, SpR).

However, for others, their concerns were significant enough to mean that continuing in silence was not an option. For these respondents who maintained that voice was impossible and ineffective, the only remaining option was exit, either leaving the job, the country or the medical profession:

[I] would rather leave [the] job, [I] do not think the system has room to change working conditions (R53, SHO).

I'm considering leaving this profession because of the conditions and I do not think the change will come quickly enough to stay working as a doctor in Ireland (R528, Intern).

### Silence for self, voice for others

While respondent doctors would not speak up about their own working conditions, many drew a distinction between speaking up for themselves and speaking up on issues directly related to patient safety:

I have raised legitimate clinical concerns in the past… [but] I have little hope for concerns regarding NCHD working conditions (R68, SpR).

No point raising concerns about our own working conditions because nothing will change. [But] I would immediately raise concerns if they related to a patient's care, and have done so in the past (R354, SHO).

While senior doctors were less likely to be silent than junior doctors, some who did choose silence drew a distinction between speaking up for themselves and speaking up for those under their supervision:

I would and have for junior doctors and nurses. For myself…I think I would not take the mental strain [of speaking up] (R349, Consultant).

## Discussion

These findings suggest that there are many factors that influence workplace silence among hospital doctors in Ireland. Among these, improvements in Irish state finances (2013–2019) have not translated into improvements in working conditions for hospital doctors ( Burke *et al.* , 2014 ; Humphries *et al.* , 2019a , b ). The survey responses suggest that this lack of improvement has bred a sense of apathy among hospital doctors and an acceptance of poor working conditions. Yet, as Edmondson (2019 , p. 26) argues, “psychological safety is *essential* to unleashing talent” across the entire workforce. Responding to workplace silence, encouraging voice without stigma and using feedback to inform health system improvements will require system and cultural solutions.

### Silence and apathy

While studies from the corporate world suggest that employees are more likely to keep silent out of fear rather than apathy ( Milliken *et al.* , 2003 ; Ryan and Oestreich, 1991 ), respondent doctors in in this study reported being more likely to remain silent about working conditions due to apathy than fear, and this was identified across all demographics of those who said they would not voice. This is similar to findings by Pope and Burnes (2013) in the UK's National Health Service (NHS), suggesting that silence in the healthcare sector might stem more from organisational deafness and stagnation than from a culture of silence. The danger for the Irish health system is that apathy among hospital doctors may further compound the problem of silence and dissuade voice ( Pinder and Harlos, 2001 ), allowing problems with working conditions to proliferate and deteriorate.

Findings from the responses suggest that junior hospital doctors in Ireland, particularly interns, feel they have less cultural capital within the profession, that their concerns are perceived as less professionally legitimate and are more silent than others. This aligns with studies of junior doctors in other countries ( Landgren *et al.* , 2016 ; Luke, 2003 ; Martinez *et al.* , 2017 ). Non-consultant hospital doctors are also on temporary contracts, and regularly change workplaces, which reflects Garsten's (1999) findings on the liminal and peripheral status of temporary workers in the workplace. If apathy towards voice becomes endemic among junior doctors, this may further entrench a status quo of silence and make it more difficult to encourage voice.

### Silence and vulnerability

Another group prone to workplace silence are those who feel vulnerable within their professional culture and worry that voice might damage their professional reputation, particularly non-consultant hospital doctors. They reported feeling vulnerable on multiple fronts: they are temporary employees, they are trainees or aspiring trainees and most aspire to achieving a secure consultant status in the Irish health system. These factors interact to encourage silence.

Competition for places in basic and higher specialist training programmes is intense, and professional connections and references from supervising consultants or clinical directors can be highly influential in the selection progress. These structures make trainees vulnerable to reputational capital and the negative consequences of voice, motivating silence; many non-consultant hospital doctor respondents felt that voicing their concerns about working conditions might harm their future career opportunities. It is also difficult for non-consultant hospital doctors to voice through professional lobby groups ( Hendrick, 2017 ): while health is one of the most prominent issues raised to the Irish government by lobby groups every year ( Register of Lobbying Ireland, 2019 ), the most vocal medical lobby groups are made up of consultant doctors only, such as the Irish Hospital Consultants Association (IHCA) and the Consultants' Committee of the Irish Medical Association (IMO).

Other factors on a demographic level, such as gender and citizenship, may further compound this vulnerability. Some female doctors and non-European doctors also felt they had less cultural capital within Irish medicine and were peripheral to those who set the norms and have legitimacy within the profession to be able to use voice safely. Many female doctors were particularly concerned about acquiring reputations that played into gender stereotypes, echoing similar findings from studies of women's workplace voice ( Babcock and Laschever, 2003 ; Meares *et al.* , 2004 ; Pinder and Harlos, 2001 ; Ryan and Oestreich, 1991 ). Although the medical profession in Ireland has become increasingly “feminized” in recent decades, McAleese (2013 , p. 12) argues that there remains “a gender bias against female doctors”, which may make some feel vulnerable and less confident in voicing concerns. Many non-European doctors were also concerned about the reputational risks of voice. The Irish health system employs almost 2,500 non-consultant hospital doctors in non-training positions, 40% of the total cohort of 6,500 non-consultant hospital doctors; over 70% of these non-trainee non-consultant hospital doctors are non-European doctors ( Medical Council of Ireland, 2018 ). Without access to training, these doctors are unable to progress their career; this may encourage them to stay silent to ensure good reputations and references and improve their chances of selection for further contracts or training posts. As part of their migration permission, these doctors are required to maintain consistent employment contracts to have their visas renewed every six months ( Department of Justice and Equality, 2015 ), which leads them to report feeling particularly vulnerable. Non-European doctors' reluctance to voice is consistent with previous studies of silence and voice, which have found that ethnic minorities in western settings may be more likely to choose silence ( Meares *et al.* , 2004 ).

### Exit, voice, loyalty or neglect

If reluctant to use voice in response to dissatisfaction with workplace conditions, as the 227 respondents in this study were, doctors are left with three other options according to Hirschman (1970) and Rusbult *et al.* (1982) – exit, loyalty or neglect. They may continue working within the system (loyalty). They may leave the Irish health system to practise medicine elsewhere, leave hospital medicine for a different speciality or for another profession, or retire (exit). Equally, they may remain within the system but neglect parts of their work or their non-work lives. Studies from the corporate world indicate that neglect of work duties is an often-used alternative to voice ( Naus *et al.* , 2007 ; Withey and Cooper, 1989 ). Professional and ethical obligations to avoid patient harm are likely to dissuade doctors from workplace neglect, but apathy and disengagement, as demonstrated in these findings, may be regarded as a form of neglect.

Many respondent doctors indicated, without prompting, that their silence led directly to plans or hopes for exit. Of the 227 silent respondents in this study, 181 mentioned considering either leaving Ireland to practice medicine or leaving medicine altogether. When voice poses a high risk, high energy cost and only a slight possibility of improvement, but exit almost guarantees improvement, exit may come to be seen as a better use of energy ( Hirschman, 1970 ). This may be especially true of early career doctors, for whom the social and emotional costs of exit are lower and the potential cost of voice is high, as Humphries *et al.* (2019a , b) suggest. Ireland already has a high rate of doctor emigration; studies suggest this is influenced by health system factors ( Byrne *et al.* , 2021 ; Humphries *et al.* , 2019a , b ). High emigration rates perpetuate more exit, as people “underestimate the effectiveness of voice when exit is dominant” ( Hirschman, 1970 , p. 125). Therefore, workplace silence and the unwillingness or inability to voice are key factors in the issue of retention for the medical workforce in Ireland.

### Working conditions and patient safety: a consistent need for voice

Enabling voice effectively for all hospital workers at an organisational level has been shown to have positive outcomes both for patients' safety ( Schwappach and Richard, 2018 ; Wilkinson *et al.* , 2018a , b ) and clinical outcomes ( Edmondson, 2019 ), as well as reducing burnout and improving retention and motivation ( Hujala and Rissanen, 2012 ; Tucker and Edmondson, 2003 ; Wilkinson *et al.* , 2018 ). Respondent doctors understood the importance of using their voices to speak up about direct patient safety issues. However, the same consideration for voice relating to their own working conditions was not evident, indicating a disconnect in doctors' minds between their own conditions at work and the care they are able to provide patients. Doctors are trained and encouraged to voice patient safety concerns ( Ward *et al.* , 2019 ), but are not similarly encouraged to voice concerns about their working conditions. Doctors may be weighing the “cost” of voice in each situation ( Hirschman 1970 ), where silence is costly for patient safety and may cause death, but it is voice that is costly for their own conditions when there may be severe professional repercussions. Yet, Avgar *et al.* (2016) and Wilkinson *et al.* (2020) provide clear links between patient safety and hospital working conditions. Likewise, West *et al.* (2018) show that patient safety and satisfaction are linked to physician well-being, which is negatively affected by silence. Initiatives to train and encourage hospital doctors to voice patient safety concerns are certainly positive steps towards high-quality healthcare, but to fully optimise patient safety, voice regarding doctors' working conditions needs to be encouraged and resolved too.

## Further research and implications

For practice, these findings offer valuable insights into the organisational, professional and cultural challenges to effective voice for hospital doctors. In particular, they indicate that many doctors across all grades are silent due to apathy and a lack of faith in the ability to change the system. Reputational risk, lack of energy and time, a perceived inability to effect change and cultural norms all discourage doctors from raising concerns about working conditions. Furthermore, many would prefer to make plans to exit rather than voice their concerns. This poses a real risk to hospital administrations and health services, especially in the COVID-19 environment where healthcare worker recruitment is highly competitive and destination countries are actively pursuing trained doctors with a mind to emigrate ( Graham-McLay, 2020 ). A “polyphonic” approach ( Hujala and Rissanen, 2012 ), where multiple voices, including critical voices, throughout all levels of the hospital workforce are sought and given space, can promote innovation and development and contribute to organisational success for the hospital and health system. Another approach may be to follow the model of the “freedom to speak up guardian” in place in the UK's NHS, which emerged in the wake of the Francis report demonstrating the horrific impact of employee silence on patient safety issues ( Hughes, 2019 ). The guardian is an independent officer within the healthcare organisation who encourages doctors to voice their concerns in confidence, escalates them confidentially to responsible stakeholders and ensures voicing doctors are aware of actions relating to their concerns; this may help provide a safe alternative route through which to voice concerns.

The policy implications of these findings reinforce the need for health systems across the world, including the Irish system, to encourage organisational and professional cultures, which are open to hearing and learning from frontline staff, without professional risk for those staff. Such a culture needs to be created and nurtured collaboratively by multiple stakeholders up, down and across the hierarchies and silos of the system ( Edmondson, 2019 ). As the 2015 Francis inquiry into the NHS found, when staff voice is ignored or seen as professionally unsafe within a health system, changes to culture and practice need to be made not only at a workplace level, but at a broader system level ( Hughes, 2019 ). Hospital administrators, health services, medical training colleges and governments need to understand and temper the factors that lead hospital doctors to remain silent about problematic working conditions, to build a safe, open system for enabling voice and implementing solutions. Not doing so poses a substantial risk to service delivery, patient safety and staff motivation and retention, particularly important in the rapidly changing frontline context of COVID-19 where apathy and fear may have severe consequences ( Bohmer *et al.* , 2020 ; Daphna-Tekoah *et al.* , 2020 ).

These findings have opened the door to many potential avenues of future research on voice among hospital doctors. Quantitatively, an in-depth exploration of whether doctors' likelihood of choosing voice or silence is linked to other factors in their evaluations and experiences of their workplaces may be fruitful. Qualitatively, asking doctors who would choose voice what factors make them feel confident to do so, or asking doctors unsure whether they would voice concerns what factors might positively or negatively influence their decision, and whether these vary by group, may shed further light on voice behaviours and motivating factors in healthcare organisations. While Humphries *et al.* (2019) touch on the role of lack of voice in emigration decisions of Irish doctors, future studies could more explicitly examine the connection between lack of voice and the pursuit of loyalty or neglect ( Allen and Tüselmann, 2009 ) amongst hospital doctors in Ireland. Studies could also explore empirically whether the psychological and physiological effects of lack of voice mentioned by Morrison and Milliken (2000) are experienced by hospital doctors. Studies of the use of social media at work as a form of workplace voice by doctors may shed light on whether silent doctors are raising concerns about working conditions in other ways, particularly the new generation of doctors ( Holland *et al.* , 2019 ). Comparative studies between Ireland and other nations may illuminate whether the factors limiting voice are specific to Irish cultural and health system phenomena, or are indicative of wider professional norms.

## Limitations

The findings of this study may not be representative of the experience of all hospital doctors for several reasons. Firstly, the low response rate, while similar to previous studies of doctors ( O'Brien *et al.* , 2019 ; Shanafelt *et al.* , 2019 ), may mean that the response trends within the cohort of respondents are not representative of the wider profession. The response rate and the self-selecting nature of participation may also mean the possibility of bias, as respondents may be made up of doctors with more negative experiences wishing to express their views anonymously. Alternatively, some doctors who experience very high levels of fear at speaking out about conditions may have declined the opportunity to participate in the survey over fears of disclosure and repercussions, despite clear messaging around the anonymity and security of responses. Open-ended responses within the survey may also not have captured the experiences of all doctors, or even all respondents, as open-ended questions often have higher non-response rates, and some respondent groups are more likely to provide responses in this format than others ( Miller and Lambert, 2014 ). Specifying a definition of “working conditions”, or providing a list of potential issues from which respondents could select, may have shed more light on the particular concerns they had but chose to remain silent about. Finally, the survey also only provided the option for silent doctors to explain why they would not raise concerns; asking voicing doctors for the reasons why they do raise concerns, or unsure doctors what factors might influence their choice, may have provided counterpoints, which strengthened this study's findings.

## Conclusion

This study has highlighted why hospital doctors may opt to remain silent about their work-related concerns. Silence places doctors at risk of self-neglect, burnout and exit. This poses risks in particular for the Irish hospital system, given its existing issues with stress and scarcity, precarity, competitive progression and heavy reliance on migrant and temporary workers. There is a need for thoughtful solutions to encourage and protect voice for those hospital doctors who are silent out of fear. There is also a need to encourage a culture of change and improvement informed by frontline voice, to re-engage those hospital doctors who are silent out of apathy. This is increasingly pertinent in world where COVID-19 continues to challenge the capacities of healthcare systems and healthcare organisations, as well as the working lives and well-being of the medical workforce. Significant changes to both the operating environment and organisational culture of hospital systems, and the professional culture of medicine, can help address the factors preventing doctors from exercising their rights to workplace voice mechanisms and raising concerns.

## Figures and Tables

**Table 1:** Demographic characteristics of silent respondents

Total respondents		227 (%)
Gender	Male	108 (48)
	Female	117 (51)
	Prefer not to say	2 (1)
Citizenship country	Irish	172 (76)
	EU country	17 (7)
	Non-EU country	38 (17)
Grade	Consultant	35 (15)
	Senior/SpR	43 (19)
	Registrar	42 (19)
	SHO	52 (23)
	Intern	55 (24)
Status	Non-consultant hospital doctor (NCHD) (yes)	192 (85)
	Locum contract (yes)	15 (7)

## References

[ref001] Allen , M. and Tüselmann , H.J. ( 2009 ), “ All powerful voice? The need to include ‘exit’, ‘loyalty’ and ‘neglect’ in empirical studies too ”, Employee Relations , Vol. 31 No. 5 , pp. 538 - 552 , doi: 10.1108/01425450910979275 .

[ref002] Anheier , H.K. , Gerhards , J. and Romo , F.P. ( 1995 ), “ Forms of capital and social structure in cultural fields: examining bourdieu's social topography ”, American Journal of Sociology , Vol. 100 No. 4 , pp. 859 - 903 , available at: www.jstor.org/stable/2782154 .

[ref003] Attree , M. ( 2007 ), “ Factors influencing nurses' decisions to raise concerns about care quality ”, Journal of Nursing Management , Vol. 15 No. 4 , pp. 392 - 402 , doi: 10.1111/j.1365-2834.2007.00679.x . 17456168

[ref004] Avgar , A.C. , Sadler , J.A. , Clark , P. and Chung , W. ( 2016 ), “ Labor–Management partnership and employee voice: evidence from the healthcare setting ”, Industrial Relations: A Journal of Economy and Society , Vol. 55 No. 4 , pp. 576 - 603 , doi: 10.1111/irel.12152 .

[ref005] Babcock , L. and Laschever , S. ( 2003 ), Women Don't Ask: Negotiation and the Gender Divide , Princeton University Press, Princeton, NJ .

[ref006] Bohmer , R. , Shand , J. , Allwood , D. , Wragg , A. and Mountford , J. ( 2020 ), “ Learning systems: managing uncertainty in the new normal of covid-19 ”, NEJM Catalyst Innovations in Care Delivery . doi: 10.1056/CAT.20.0318 .

[ref007] Bourdieu , P. ( 1982 ), “ The forms of capital ”, in Richardson , J.G. (Ed.), Handbook of Theory and Research for the Sociology of Education , Greenwood, Westport, CT , pp. 241 - 258 .

[ref008] Braun , V. and Clarke , V. ( 2006 ), “ Using thematic analysis in psychology ”, Qualitative Research in Psychology , Vol. 3 No. 2 , pp. 77 - 101 , doi: 10.1191/1478088706qp063oa .

[ref009] Brugha , R. , McAleese , S. , Dicker , P. , Tyrrell , E. , Thomas , S. , Normand , C. and Humphries , N. ( 2016 ), “ Passing through – reasons why migrant doctors in Ireland plan to stay, return home or migrate onwards to new destination countries ”, Human Resources for Health , Vol. 14 No. 1 , p. 35 , doi: 10.1186/s12960-016-0121-z . 27381409 PMC4943478

[ref010] Burke , S. , Thomas , S. , Barry , S. and Keegan , C. ( 2014 ), “ Indicators of health system coverage and activity in Ireland during the economic crisis 2008-2014 - from ‘more with less' to ‘less with less' ”, Health Policy , Vol. 117 No. 3 , pp. 275 - 278 , doi: 10.1016/j.healthpol.2014.07.001 . 25082466

[ref011] Burke , S. , Barry , S. , Siersbaek , R. , Johnston , B. , Ní Fhallúin , M. and Thomas , S. ( 2018 ), “ Sláintecare – a ten-year plan to achieve universal healthcare in Ireland ”, Health Policy , Vol. 122 No. 12 , pp. 1278 - 1282 , doi: 10.1016/j.healthpol.2018.05.006 . 29843901

[ref012] Byrne , J.P. , Conway , E. , McDermott , A.M. , Matthews , A. , Prihodova , L. , Costello , R.W. and Humphries , N. ( 2021 ), “ How the organisation of medical work shapes the everyday work experiences underpinning doctor migration trends: the case of Irish-trained emigrant doctors in Australia ”, Health Policy , Vol. 125 No. 4 , pp. 467 - 473 , doi: 10.1016/j.healthpol.2021.01.002 . 33551205

[ref013] Crowe , S. , Clarke , N. and Brugha , R. ( 2017 ), “ ‘You do not cross them’: hierarchy and emotion in doctors' narratives of power relations in specialist training ”, Social Science and Medicine , Vol. 186 , pp. 70 - 77 , doi: 10.1016/j.socscimed.2017.05.048 . 28587868

[ref014] Daphna-Tekoah , S. , Megadasi Brikman , T. , Scheier , E. and Balla , U. ( 2020 ), “ Listening to hospital personnel's narratives during the COVID-19 outbreak ”, International Journal of Environmental Research and Public Health , Vol. 17 No. 17 , p. 6413 , doi: 10.3390/ijerph17176413 . PMC7503987 32899163

[ref015] Department of Justice and Equality ( 2015 ), Non-EEA Doctors - Immigration and Working Arrangements , Department of Justice and Equality , available at: http://www.inis.gov.ie/en/INIS/Pages/ARRANGEMENTS%20FOR%20NON-EEA%20DOCTORS%20WORKING%20IN%20THE%20STATE ( accessed 9 February 2021 ).

[ref016] Dixon-Woods , M. , Campbell , A. , Martin , G. , Willars , J. , Tarrant , C. , Aveling , E.L. , Sutcliffe , K. , Clements , J. , Carlstrom , M. and Pronovost , P. ( 2019 ), “ Improving employee voice about transgressive or disruptive behavior: a case study ”, Academic Medicine , Vol. 94 No. 4 , pp. 579 - 585 , doi: 10.1097/acm.0000000000002447 . 30211753 PMC6330059

[ref017] Edmondson , A.C. ( 2019 ), The Fearless Organization: Creating Psychological Safety in the Workplace for Learning, Innovation, and Growth , Wiley, Hoboken, NJ .

[ref018] Fereday , J. and Muir-Cochrane , E. ( 2006 ), “ Demonstrating rigor using thematic analysis: a hybrid approach of inductive and deductive coding and theme development ”, International Journal of Qualitative Methods , Vol. 5 No. 1 , pp. 80 - 92 , doi: 10.1177/160940690600500107 .

[ref019] Garsten , C. ( 1999 ), “ Betwixt and between: temporary employees as liminal subjects in flexible organizations ”, Organization Studies , Vol. 20 No. 4 , pp. 601 - 617 , doi: 10.1177/0170840699204004 .

[ref020] Graham-McLay , C. ( 2020 ), I Love This Country': US Doctors Head to New Zealand as Cure for America's Ills , The Guardian , available at: https://www.theguardian.com/world/2020/oct/16/i-love-this-country-us-doctors-head-to-new-zealand-as-cure-for-americas-ills .

[ref021] Hayes , B. , Prihodova , L. , Walsh , G. , Doyle , F. and Doherty , S. ( 2019 ), “ Doctors don't Do-little: a national cross-sectional study of workplace well-being of hospital doctors in Ireland ”, BMJ Open , Vol. 9 No. 3 , e025433 , doi: 10.1136/bmjopen-2018-025433 . PMC6429874 30853661

[ref022] Helmreich , R.L. and Merritt , A.C. ( 1998 ), Culture at Work in Aviation and Medicine: National, Organizational and Professional Influences , Ashgate, Brookfield, VT .

[ref023] Hendrick , L. ( 2017 ), “ Diagnosis for the future ”, Accounting and Business , pp. 12 - 15 , June 2017, available at: https://www.accaglobal.com/content/dam/ACCA_Global/Members/AB/2017/June/AB-IE-June-2017.pdf .

[ref024] Hirschman , A.O. ( 1970 ), Exit, Voice, and Loyalty: Responses to Decline in Firms, Organizations, and States , Harvard University Press, Cambridge, MA .

[ref025] Hobson , B. ( 2013 ), Worklife Balance: The Agency and Capabilities Gap , Oxford University Press, Oxford .

[ref026] Holland , P. , Cooper , B. and Hecker , R. ( 2019 ), “ Social media at work: a new form of employee voice? ”, in Holland , P. , Teicher , J. and Donaghey , J. (Eds), Employee Voice at Work , Springer, Singapore , pp. 73 - 89 , doi: 10.1007/978-981-13-2820-6_4 .

[ref027] Hughes , H. ( 2019 ), “ Freedom to speak up - the role of freedom to speak up guardians and the National Guardian's Office in England ”, Future Healthcare Journal , Vol. 6 No. 3 , pp. 186 - 189 , doi: 10.7861/fhj.2019-0031 . 31660523 PMC6798024

[ref028] Hujala , A. and Rissanen , S. ( 2012 ), “ Discursive construction of polyphony in healthcare management ”, Journal of Health Organization and Management , Vol. 26 No. 1 , pp. 118 - 136 , doi: 10.1108/14777261211211124 . 22524102

[ref029] Humphries , N. , McAleese , S. , Matthews , A. and Brugha , R. ( 2015 ), “ 'Emigration is a matter of self-preservation. The working conditions . . . are killing us slowly': qualitative insights into health professional emigration from Ireland ”, Human Resources for Health , Vol. 13 No. 1 , p. 35 , doi: 10.1186/s12960-015-0022-6 . 25981629 PMC4437248

[ref030] Humphries , N. , Connell , J. , Negin , J. and Buchan , J. ( 2019a ), “ Tracking the leavers: towards a better understanding of doctor migration from Ireland to Australia 2008–2018 ”, Human Resources for Health , Vol. 17 No. 1 , p. 36 , doi: 10.1186/s12960-019-0365-5 . 31138211 PMC6540407

[ref031] Humphries , N. , McDermott , A.M. , Conway , E. , Byrne , J.P. , Prihodova , L. , Costello , R. and Matthews , A. ( 2019b ), “ 'Everything was just getting worse and worse': deteriorating job quality as a driver of doctor emigration from Ireland ”, Human Resources for Health , Vol. 17 No. 1 , p. 97 , doi: 10.1186/s12960-019-0424-y . 31815621 PMC6902557

[ref032] Humphries , N. , McDermott , A.M. , Creese , J. , Matthews , A. , Conway , E. and Byrne , J.P. ( 2020 ), “ Hospital doctors in Ireland and the struggle for work-life balance ”, European Journal of Public Health , Vol. 30 No. 4 , pp. 432 - 435 , doi: 10.1093/eurpub/ckaa130 . 32894279 PMC7526767

[ref033] Jones , A. and Kelly , D. ( 2014 ), “ Deafening silence? Time to reconsider whether organisations are silent or deaf when things go wrong ”, BMJ Quality and Safety , Vol. 23 No. 9 , pp. 709 - 713 , doi: 10.1136/bmjqs-2013-002718 . 25015116

[ref034] Keller , E.J. , Giafaglione , B. , Chrisman , H.B. , Collins , J.D. and Vogelzang , R.L. ( 2019 ), “ The growing pains of physician-administration relationships in an academic medical center and the effects on physician engagement ”, PloS One , Vol. 14 No. 2 , e0212014 , doi: 10.1371/journal.pone.0212014 . 30759151 PMC6373942

[ref035] Kennelly , B. , O'Callaghan , M. , Coughlan , D. , Cullinan , J. , Doherty , E. , Glynn , L. , Moloney , E. and Queally , M. ( 2020 ), “ The COVID-19 pandemic in Ireland: an overview of the health service and economic policy response ”, Health Policy and Technology , Vol. 9 No. 4 , pp. 419 - 429 , doi: 10.1016/j.hlpt.2020.08.021 . 32923355 PMC7480279

[ref036] Kenny , K. ( 2019 ), Whistleblowing: Toward a New Theory , Harvard University Press, Cambridge, MA .

[ref081] Knoll , M. and Redman , T. ( 2016 ), “ Does the presence of voice imply the absence of silence? The necessity to consider employees' affective attachment and job engagement ”, Human Resources Management , Vol. 55 No. 5 , pp. 829 - 844 , doi: 10.1002/hrm.21744 .

[ref037] Landgren , R. , Alawadi , Z. , Douma , C. , Thomas , E.J. and Etchegaray , J. ( 2016 ), “ Barriers of pediatric residents to speaking up about patient safety ”, Hospital Pediatrics , Vol. 6 No. 12 , pp. 738 - 743 , doi: 10.1542/hpeds.2016-0042 . 27909093

[ref038] Lipset , S.M. , Meltz , N.M. and Gomez , R. ( 2004 ), “ Unions among professionals and other white-collar workers in the United States ”, in Lipset , S.M. , Meltz , N.M. and Gomez , R. (Eds), The Paradox of American Unionism: Why Americans like Unions More than Canadians Do, but Join Much Less , ILR Press, Ithaca, NY , pp. 118 - 144 .

[ref039] Lock , M.J. , Stephenson , A.L. , Branford , J. , Roche , J. , Edwards , M.S. and Ryan , K. ( 2017 ), “ Voice of the Clinician: the case of an Australian health system ”, Journal of Health Organization and Management , Vol. 31 No. 6 , pp. 665 - 678 , doi: 10.1108/JHOM-05-2017-0113 . 29034826 PMC5868555

[ref040] Luke , H. ( 2003 ), Medical Education and Sociology of Medical habitus: ‘It's Not about the Stethoscope!’ , Kluwer Academic Publishers, Dordrecht .

[ref041] Mahood , S.C. ( 2011 ), “ Medical education: beware the hidden curriculum ”, Canadian family physician Medecin de famille canadien , Vol. 57 No. 9 , pp. 983 - 985 , available at: https://www.cfp.ca/content/57/9/983.long . 21918135 PMC3173411

[ref042] Martin , G.P. , Aveling , E.L. , Campbell , A. , Tarrant , C. , Pronovost , P.J. , Mitchell , I. , Dankers , C. , Bates , D. and Dixon-Woods , M. ( 2018 ), “ Making soft intelligence hard: a multi-site qualitative study of challenges relating to voice about safety concerns ”, BMJ Quality and Safety , Vol. 27 No. 9 , pp. 710 - 717 , doi: 10.1136/bmjqs-2017-007579 . PMC6109252 29459365

[ref043] Martinez , W. , Lehmann , L.S. , Thomas , E.J. , Etchegaray , J.M. , Shelburne , J.T. , Hickson , G.B. , Brady , D.W. , Schleyer , A.M. , Best , J.A. , May , N.B. and Bell , S.K. ( 2017 ), “ Speaking up about traditional and professionalism-related patient safety threats: a national survey of interns and residents ”, BMJ Quality and Safety , Vol. 26 No. 11 , pp. 869 - 880 , doi: 10.1136/bmjqs-2016-006284 . 28442609

[ref044] McAleese , S. ( 2013 ), The Feminisation of Medicine: A Qualitative Study of the Career Experiences of Female Doctors and Implications For Human Resources Management in Ireland , PhD Thesis , Royal College of Surgeons in Ireland, Dublin .

[ref045] McGowan , Y. , Humphries , N. , Burke , H. , Conry , M. and Morgan , K. ( 2013 ), “ Through doctors' eyes: a qualitative study of hospital doctor perspectives on their working conditions ”, British Journal of Health Psychology , Vol. 18 No. 4 , pp. 874 - 891 , doi: 10.1111/bjhp.12037 . 23480457

[ref046] McNicholas , F. , Sharma , S. , Oconnor , C. and Barrett , E. ( 2020 ), “ Burnout in consultants in child and adolescent mental health services (CAMHS) in Ireland: a cross-sectional study ”, BMJ Open , Vol. 10 No. 1 , e030354 , doi: 10.1136/bmjopen-2019-030354 . PMC7045151 31959602

[ref047] Meares , M.M. , Oetzel , J.G. , Torres , A. , Derkacs , D. and Ginossar , T. ( 2004 ), “ Employee mistreatment and muted voices in the culturally diverse workplace ”, Journal of Applied Communication Research , Vol. 32 No. 1 , pp. 4 - 27 , doi: 10.1080/0090988042000178121 .

[ref048] Medical Council of Ireland ( 2018 ), “ Medical workforce intelligence report: 2018 annual registration retention and voluntary registration withdrawal surveys ”, available at: https://medicalcouncil.ie/news-and-publications/publications/medical-workforce-intelligence-report-2018-annual-retention-summary.pdf .

[ref049] Miller , A.L. and Lambert , A.D. ( 2014 ), “ Open-ended survey questions: item nonresponse nightmare or qualitative data dream ”, Survey Practice , Vol. 7 No. 5 , pp. 1 - 11 , doi: 10.29115/SP-2014-0024 .26451335

[ref050] Milliken , F.J. , Morrison , E.W. and Hewlin , P.F. ( 2003 ), “ An exploratory study of employee silence: issues that employees don't communicate upward and why ”, Journal of Management Studies , Vol. 40 No. 6 , pp. 1453 - 1476 , doi: 10.1111/1467-6486.00387 .

[ref051] Monrouxe , L.V. ( 2010 ), “ Identity, identification and medical education: why should we care? ”, Medical Education , Vol. 44 No. 1 , pp. 40 - 49 , doi: 10.1111/j.1365-2923.2009.03440.x . 20078755

[ref052] Morrison , E. ( 2011 ), “ Employee voice behavior: integration and directions for future research ”, The Academy of Management Annals , Vol. 5 , pp. 373 - 412 , doi: 10.1080/19416520.2011.574506 .

[ref053] Morrison , E. and Milliken , F. ( 2000 ), “ Organizational silence: a barrier to change and development in a pluralistic world ”, Academy of Management Review , Vol. 25 No. 4 , pp. 706 - 725 , doi: 10.2307/259200 .

[ref054] Naus , F. , van Iterson , A. and Roe , R. ( 2007 ), “ Organizational cynicism: extending the exit, voice, loyalty, and neglect model of employees' responses to adverse conditions in the workplace ”, Human Relations , Vol. 60 No. 5 , pp. 683 - 718 , doi: 10.1177/0018726707079198 .

[ref055] O'Brien , S. , Prihodova , L. , Heffron , M. and Wright , P. ( 2019 ), “ Physical activity counselling in Ireland: a survey of doctors' knowledge, attitudes and self-reported practice ”, BMJ Open Sport and Exercise Medicine , Vol. 5 No. 1 , e000572 , doi: 10.1136/bmjsem-2019-000572 . PMC6677951 31423324

[ref056] O'Dea , B. , O'Connor , P. , Lydon , S. and Murphy , A.W. ( 2017 ), “ Prevalence of burnout among Irish general practitioners: a cross-sectional study ”, Irish Journal of Medical Science , Vol. 186 No. 2 , pp. 447 - 453 , doi: 10.1007/s11845-016-1407-9 . 26803315

[ref057] Pinder , C.C. and Harlos , K.P. ( 2001 ), “ Employee silence: quiescence and acquiescence as responses to perceived injustice ”, Research in Personnel and Human Resources Management , Vol. 20 , pp. 331 - 370 , doi: 10.1016/S0742-7301(01)20007-3 .

[ref058] Pope , R. and Burnes , B. ( 2013 ), “ A model of organisational dysfunction in the NHS ”, Journal of Health Organization and Management , Vol. 27 No. 6 , pp. 676 - 697 , doi: 10.1108/JHOM-10-2012-0207 . 24422253

[ref059] Popping , R. ( 2015 ), “ Analyzing open-ended questions by means of text analysis procedures ”, BMS: Bulletin of Sociological Methodology / Bulletin de Méthodologie Sociologique , Vol. 128 No. 1 , pp. 23 - 39 , doi: 10.1177/0759106315597389 .

[ref060] Pyman , A. , Cooper , B. , Teicher , J. and Holland , P. ( 2006 ), “ A comparison of the effectiveness of employee voice arrangements in Australia ”, Industrial Relations Journal , Vol. 37 No. 5 , pp. 543 - 559 , doi: 10.1111/j.1468-2338.2006.00419.x .

[ref061] Register of Lobbying Ireland ( 2019 ), “ Regulation of lobbying in 2019: annual report ”, available at: https://www.lobbying.ie/media/6270/regulation-of-lobbying-annual-report-2019-final-web.pdf .

[ref062] Rusbult , C.E. , Zembrodt , I.M. and Gunn , L.K. ( 1982 ), “ Exit, voice, loyalty, and neglect: responses to dissatisfaction in romantic involvements ”, Journal of Personality and Social Psychology , Vol. 43 No. 6 , pp. 1230 - 1242 , doi: 10.1037/0022-3514.43.6.1230 .

[ref063] Ryan , K. and Oestreich , D.K. ( 1991 ), Driving Fear Out of the Workplace: How to Overcome the Invisible Barriers to Quality, Productivity, and Innovation , Jossey Bass, San Francisco .

[ref064] Schwappach , D. and Richard , A. ( 2018 ), “ Speak up-related climate and its association with healthcare workers' speaking up and withholding voice behaviours: a cross-sectional survey in Switzerland ”, BMJ Quality and Safety , Vol. 27 No. 10 , pp. 827 - 835 , doi: 10.1136/bmjqs-2017-007388 . PMC6166598 29572300

[ref065] Shanafelt , T.D. , West , C.P. , Sinsky , C. , Trockel , M. , Tutty , M. , Satele , D.V. , Carlasare , L.E. and Dyrbye , L.N. ( 2019 ), “ Changes in burnout and satisfaction with work-life integration in physicians and the general US working population between 2011 and 2017 ”, Mayo Clinic Proceedings , Vol. 94 No. 9 , pp. 1681 - 1694 , doi: 10.1016/j.mayocp.2018.10.023 . 30803733

[ref066] Tucker , A.L. and Edmondson , A.C. ( 2003 ), “ Why hospitals don't learn from failures: organizational and psychological dynamics that inhibit system change ”, California Management Review , Vol. 45 No. 2 , pp. 55 - 72 , doi: 10.2307/41166165 .

[ref067] Turner , B. ( 2018 ), “ Putting Ireland's health spending into perspective ”, The Lancet , Vol. 391 No. 10123 , pp. 833 - 834 , doi: 10.1016/S0140-6736(18)30461-6 . 29508738

[ref068] Van Dyne , L. , Ang , S. and Botero , I.C. ( 2003 ), “ Conceptualizing employee silence and employee voice as multidimensional constructs ”, Journal of Management Studies , Vol. 40 No. 6 , pp. 1359 - 1392 , doi: 10.1111/1467-6486.00384 .

[ref069] Ward , M. , Ní Shé , É. , De Brún , A. , Korpos , C. , Hamza , M. , Burke , E. , Duffy , A. , Egan , K. , Geary , U. , Holland , C. , O'Grady , J. , Robinson , K. , Smith , A. , Watson , A. and McAuliffe , E. ( 2019 ), “ The co-design, implementation and evaluation of a serious board game 'PlayDecide patient safety' to educate junior doctors about patient safety and the importance of reporting safety concerns ”, BMC Medical Education , Vol. 19 No. 1 , p. 232 , doi: 10.1186/s12909-019-1655-2 . 31238936 PMC6593521

[ref070] Wear , D. and Zarconi , J. ( 2010 ), “ Challenging the profession: mentoring for fearlessness ”, in Humphrey , H.J. (Ed.), Mentoring in Academic Medicine , American College of Physicians, Philadelphia, PA , pp. 51 - 66 .

[ref071] West , C.P. , Dyrbye , L.N. and Shanafelt , T.D. ( 2018 ), “ Physician burnout: contributors, consequences and solutions ”, Journal of Internal Medicine , Vol. 283 No. 6 , pp. 516 - 529 , doi: 10.1111/joim.12752 . 29505159

[ref072] Wilkinson , A. , Dundon , T. , Marchington , M. and Ackers , P. ( 2004 ), “ Changing patterns of employee voice: case studies from the UK and Republic of Ireland ”, Journal of Industrial Relations , Vol. 43 No. 3 , pp. 298 - 323 , doi: 10.1111/j.0022-1856.2004.00143.x .

[ref073] Wilkinson , A. , Townsend , K. , Graham , T. and Muurlink , O. ( 2015 ), “ Fatal consequences: an analysis of the failed employee voice system at the Bundaberg Hospital ”, Asia Pacific Journal of Human Resources , Vol. 53 No. 3 , pp. 265 - 280 , doi: 10.1111/1744-7941.12061 .

[ref074] Wilkinson , A. , Gollan , P.J. , Kalfa , S. and Xu , Y. ( 2018a ), “ Voices unheard: employee voice in the new century ”, The International Journal of Human Resource Management , Vol. 29 No. 5 , pp. 711 - 724 , doi: 10.1080/09585192.2018.1427347 .

[ref075] Wilkinson , A. , Muurlink , O. , Awan , N. and Townsend , K. ( 2018b ), “ HRM and the health of hospitals ”, Health Services Management Research , Vol. 32 No. 2 , pp. 89 - 102 , doi: 10.1177/0951484818805369 . 30376384

[ref076] Wilkinson , A. , Avgar , A. , Barry , M. and Mowbray , P. ( 2020 ), “ Voice bundles in healthcare: the reciprocal relationship between worker and patient-focused voice ”, in Wilkinson , A. , Donaghey , J. , Dundon , T. and Freeman , R.B. (Eds), Handbook of Research on Employee Voice , Edward Elgar, Cheltenham , pp. 556 - 565 , doi: 10.4337/9781788971188.00042 .

[ref077] Wiskow , C. ( 2017 ), The Role of Decent Work in the Health Sector in *Health Employment and Economic Growth: An Evidence Base* , World Health Organisation , pp. 363 - 386 , available at: https://www.who.int/hrh/resources/WHO-HLC-Report_web.pdf .

[ref078] Withey , M.J. and Cooper , W.H. ( 1989 ), “ Predicting exit, voice, loyalty, and neglect ”, Administrative Science Quarterly , Vol. 34 No. 4 , pp. 521 - 539 , doi: 10.2307/2393565 .

